# Robot-Assisted Retroperitoneoscopic Surgery for Synchronous Contralateral Ureteral Metastasis of Renal-Cell Carcinoma

**DOI:** 10.1089/cren.2015.0023

**Published:** 2015-12-01

**Authors:** Kau Han Lee, Wei-Hong Lai, Allen Wen-Shien Chiu, Chih-Cheng Lu, Steven Kuan-Hua Huang

**Affiliations:** ^1^ Division of Urology, Department of Surgery, Chi Mei Medical Center, Tainan, Taiwan.; ^2^ Division of Urology, Department of Surgery, Ditmanson Medical Foundation, Chia-Yi Christian Hospital, Chia-Yi City, Taiwan.; ^3^ School of Medicine, National Yang-Ming University, Taipei, Taiwan.; ^4^ Division of Urology, Department of Surgery, Chi Mei Medical Center, Liouying, Tainan, Taiwan.; ^5^ Division of Uro-Oncology, Department of Surgery, Chi Mei Medical Center, Tainan, Taiwan.; ^6^ College of Human Ecology, Chia-Nan University of Pharmacy & Science, Tainan, Taiwan.

## Abstract

Renal-cell carcinoma (RCC) with synchronous metastasis to contralateral ureter is extremely rare with only four cases reported in the literature. We report a case of synchronous metastatic RCC to the contralateral ureter with effective robot-assisted retroperitoneoscopic nephron-sparing surgery that leads to favorable oncologic and functional outcome.

## Introduction

Metastasis of renal-cell carcinoma (RCC) to ureter is extremely rare. In our review, a total of 54 cases had been reported about RCC with ureteral metastasis and only 11 patients out of them developed contralateral ureteral metastasis. As urologists, cancer control with preservation of renal function was important when treating RCC. Herein, we presented a patient having RCC with synchronous contralateral ureteral metastasis that was effectively managed by concomitant robot-assisted retroperitoneoscopic partial nephrectomy and segmental resection of the ureter with ureteroureterostomy.

## Case Reports

**FIG. 1.**
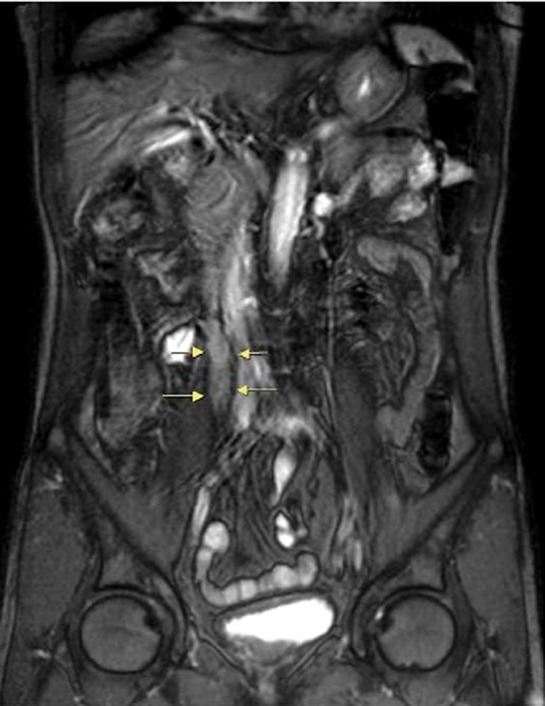
A 3.5 cm nodular lesion over right upper third ureter (arrow).

**FIG. 2.**
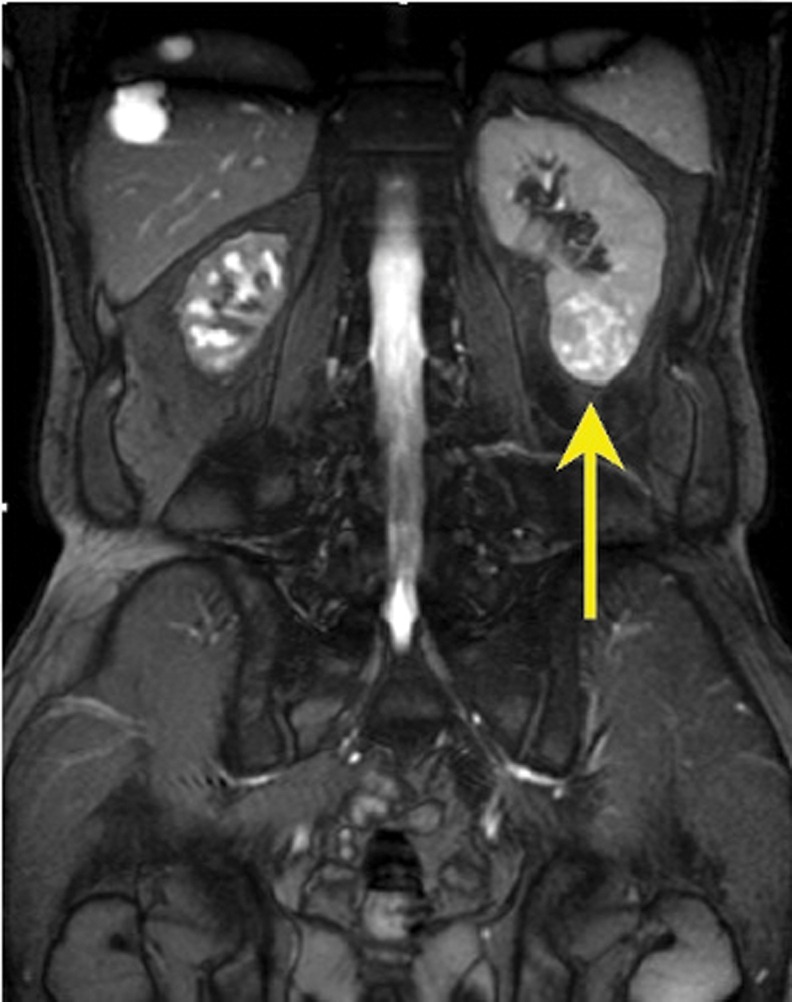
A 3 cm nodular lesion over left lower pole kidney (arrow).

**FIG. 3.**
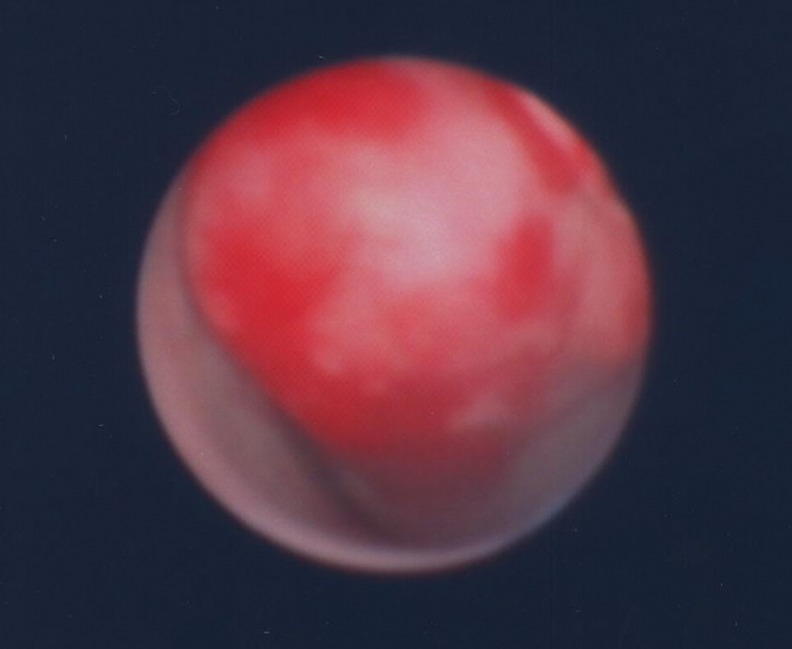
A nodular-like mass was occupied over right upper third ureter.

**FIG. 4.**
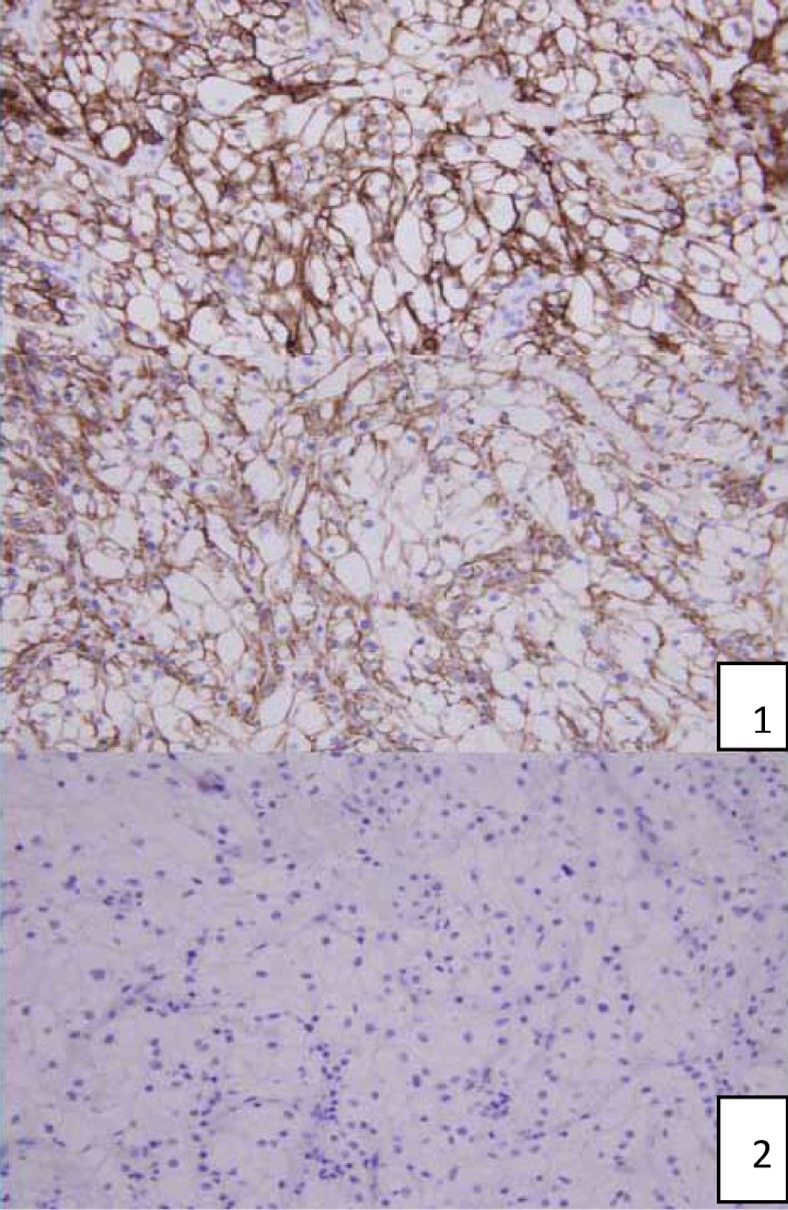
Endoscopic biopsy from right ureteral tumor: immunohistochemical revealed (1) positive CD10 and vimentin and (2) negative CK7 and CK20.

A 62-year-old healthy Taiwanese man suffered from intermittent right flank pain for 1 month. He visited a local hospital at first, where intravenous pyelography revealed an obstructive lesion over right upper third ureter. He was referred to our outpatient department for further management. Impaired renal function was noted with serum creatinine (Cr) showing 1.63 mg/dL and a renal function test (glomerular filtration rate) showing diminished renal function on both sides with severely advanced on the right (left kidney: 28 mL/minute, right kidney: 6 mL/minute). Abdominal MRI revealed a 3.5 cm tumor over right upper third ureter with moderate hydronephrosis (Fig. 1). Besides, a 3 cm nodular lesion was also detected over left lower pole kidney in favor of RCC (Fig. 2). Thus, the diagnostic ureterorenoscopy was performed (Fig. 3), and a pathology report from endoscopic biopsy to right ureter tumor presented as metastatic RCC (Fig. 4). After extensive metastatic evaluations, the ureteral lesion was believed to be solitary metastasis. With the intention of preserving renal function as well as the principle of RCC management, robot-assisted left retroperitoneoscopic partial nephrectomy and right retroperitoneoscopic segmental resection of ureter and ureteroureterostomy were performed.

The patient was positioned in a right decubitus position initially for robot-assisted left retroperitoneoscopic partial nephrectomy. The robotic surgery was performed with four robotic ports and one assistant port. The retroperitoneal space was created using balloon dilator at first, and then it was expanded with a blunt laparoscopic forceps to allow the introduction of the fourth robotic port and assistant port. The tumor was excised after it was confirmed by the intracorporeal ultrasound and the renal pedicle was controlled with bulldog clamp. The renal defect was sutured in two layers with 3-0 and 2-0 monocryl sutures. The bulldog clamp was released once the inner layer had been sutured. FLOSEAL was used for coverage of the renal defect. The surgery lasted for 109 minutes. The warm ischemic time was 19 minutes and the blood loss was 200 mL. After the partial nephrectomy was finished, the patient was placed in a left decubitus position for the second procedure. The right retroperitoneal space was created using the same method as previously mentioned. After the right ureter was identified, two stay sutures were put at the proximal and distal end of the tumor for reducing the tension that would be produced by the ureteroureterostomy. The ureteral tumor was excised smoothly and 3-0 monocryl suture was used to reapproximate the ureter. Both the proximal and distal cut ends of the tumors were sent for frozen section, which was free of tumor. The second procedure lasted for 86 minutes and the blood loss was minimal. The patient's postoperative recovery was uneventful and he was discharged on postoperative day 4. The left renal tumor and right upper third ureteral lesion were confirmed to be clear cell type RCC with Fuhrman grade 2/4 and clear surgical margin. Sunitinib was prescribed due to metastatic RCC. The patient's renal function was stable and he remained disease free during the 1-year follow-up until now.

## Discussion

It is reported that approximately one-third of patients with RCC would present with metastases. Several atypical presentations and rare metastatic sites have been reviewed in the literature.^1^ RCC with synchronous ureteral metastasis is extremely rare with just four cases having been reported.^2^ Currently, there is still no standard management. Greco reported that improved cancer-specific survival has been seen in metastatic RCC cases that had complete surgical metastasectomy.^3^ Therefore, it had been recommended as the principle of treating metastatic RCC. Our patient received concomitant partial nephrectomy with complete excision of the metastatic lesion using the robotic device. It not only provides favorable oncologic and functional outcome but also decreases the surgical morbidity. Besides, we could learn from this case that detailed evaluation of the patient with suspected ureteral lesion is crucial as early detection of the tumor leads to better management in such case.^4^

## Conclusion

In our review, this is the fifth case presented as RCC with synchronous contralateral ureteral metastasis. Robot-assisted retroperitoneoscopic partial nephrectomy with segmental resection of contralateral ureter and reconstruction with ureteroureterostomy seems effective as surgical treatment. It has a satisfied result in both the oncologic and functional outcomes.

## Abbreviations Used

MRI: magnetic resonance imaging
RCC: renal-cell carcinoma
